# Relationship of Tumor Localization and Lipid Parameters with Survival in Patients with Colorectal Cancer

**DOI:** 10.3390/jcm14041302

**Published:** 2025-02-15

**Authors:** Özlem Nuray Sever, Tuğba Başoğlu, Sedat Yıldırım

**Affiliations:** 1 Department of Medical Oncology, Medical Park Bahcelievler Hospital, Istanbul 34180, Türkiye; 2 Department of Medical Oncology, Health Science University, Kartal Dr. Lütfi Kirdar City Hospital, Istanbul 34865, Türkiye; basoglutugba@gmail.com (T.B.); rezansedat@hotmail.com (S.Y.)

**Keywords:** colorectal cancer, lipid parameters, tumor localization, survival

## Abstract

**Background**: Colorectal cancer (CRC) remains a global health challenge. Metabolic disorders, including dyslipidemia, have been linked to CRC progression, yet the relationship between lipid profiles, tumor location, and survival outcomes remains controversial. This study investigates the association between blood lipid parameters, tumor localization, and survival in CRC patients. **Methods**: A retrospective analysis was conducted on 126 CRC patients diagnosed between 2017 and 2024 at Kartal Dr. Lütfi Kırdar City Hospital. Patients with comorbidities affecting lipid metabolism or who were on lipid-lowering drugs were excluded. Clinical, pathological, and lipid data, including total cholesterol (TC), triglycerides (TGs), low-density lipoprotein cholesterol (LDL-C), high-density lipoprotein cholesterol (HDL-C), and carcinoembryonic antigen (CEA), were analyzed. Tumor location was categorized as right-sided or left-sided. Overall survival (OS) was evaluated with a statistical analysis using Kaplan–Meier and Cox regression models. **Results**: Higher HDL-C levels and a lower TC/HDL-C ratio were significantly associated with improved OS (*p*: 0.004 and *p*: 0.016, respectively). This relationship remained significant in early- and advanced-stage disease (*p*: 0.04 for HDL-C and *p*: 0.03 for TC/HDL-C). In patients with tumors located in the right colon, LDL-C levels of 150 mg/dL and below were found to be statistically positively correlated with overall survival, while in patients with tumors located in the left colon, HDL-C levels of 45 mg/dL and above and TC/HDL-C levels of 4.16 and above were found to be statistically positively correlated with overall survival. A multivariate analysis confirmed that age, stage, HDL-C, and TC/HDL-C were independent predictors of OS. **Conclusions**: Our study highlights the potential role of lipid profiles, particularly HDL-C and the TC/HDL-C ratio, as prognostic factors in CRC. Further research, including molecular and genetic analyses, is needed to better understand the mechanisms underlying the relationship between lipid metabolism and CRC progression.

## 1. Introduction

According to the World Health Organization’s GLOBOCAN 2022 database [1], colorectal cancer (CRC) is the third most commonly diagnosed cancer in men and the second most common in women globally. In the United States, CRC incidence rates had been declining by about 2% per year, but this decrease slowed to approximately 1.2% per year during the 2014–2018 period [2]. In most other Western countries, the incidence remained stable or slightly increased during this period. In contrast, CRC incidence rates rapidly increased in several historically low-risk regions, including Spain, East Asia, and Eastern Europe [3,4].

Although advancements in screening and surgical techniques over the past few decades have improved the 5-year survival rate in high-income countries [5], recurrence remains a common and challenging issue [6]. A gradual shift towards right-sided or proximal colon cancers has been observed both in the United States [7,8] and internationally [9,10], with the largest relative increase seen in cecal cancers. In recent years, the laterality of primary tumors has been considered a predictive factor for the prognosis of colon cancer patients. Right-sided cancers have been associated with lower survival rates in metastatic colon cancer patients [11,12,13,14,15].

A known risk factor for colorectal cancer is metabolic syndrome, which encompasses various metabolic disorders such as obesity, hypertension, hyperglycemia, and dyslipidemia [16]. Dyslipidemia is characterized by reduced high-density lipoprotein cholesterol (HDL-C) levels and increased low-density lipoprotein cholesterol (LDL-C) and triglyceride levels [16]. LDL-C has been shown to induce inflammation and tumor progression in human colorectal cancer cells through the activation of reactive oxygen species (ROS) and the MAPK signaling pathway [17].

Despite convincing experimental evidence, previous epidemiological studies have reported conflicting findings regarding the relationships between total cholesterol, LDL-C, HDL-C, triglycerides, and colorectal cancer risk [18,19,20,21,22,23,24,25]. Several factors, including relatively small sample sizes and reverse causality (i.e., changes in lipid levels induced by colorectal cancer development), may contribute to these inconsistencies. The number of colorectal cancer cases in previous studies ranged from 102 to 4984, with most studies involving fewer than 500 cases [18,19]. Additionally, many of these studies did not account for the regular use of cholesterol-lowering medications, which could bias lipid–cancer relationships since lipid levels measured at the time of diagnosis do not reflect long-term exposure.

Increased LDL-C has been proposed as a factor related to cancer progression, including colon cancer [26], while increased HDL-C appears to be inversely related to progression. For example, a study of ovarian cancer patients found that improvements in HDL-C levels post-surgery were associated with cancer remission [27]. Studies have also found relationships between triglycerides (TGs), total cholesterol, and CRC stage [28], and a higher LDL-C/HDL-C ratio has been associated with poor prognosis in CRC [29]. However, the exact role of dyslipidemia in CRC remains controversial [30,31,32,33].

Our study aims to investigate the relationship between blood lipid parameters, tumor location, and survival in patients diagnosed with CRC.

## 2. Materials and Methods

### 2.1. Study Design and Data Collection

Patients diagnosed with colorectal cancer and followed up and treated between 2017 and 2024 were retrospectively analyzed. Patients whose fasting blood sugar, total cholesterol, triglycerides, LDL-C, HDL-C, and CEA levels were measured in the last 3 months before starting treatment for colorectal cancer were included in the study. Patients with a history of previous or concurrent cancer, comorbidities related to lipid metabolism disorders (diabetes, hypothyroidism), or a history of lipid-lowering medication use were excluded from the study. Detailed clinical and pathological information, including age, gender, smoking status, tumor stage, pathological type, tumor location, and TNM stage, of the 126 patients included in the study were obtained from the patients’ medical records. Laboratory values for TC, TGs, LDL-C, HDL-C, and CEA were accepted as cut-off points (upper limit of normal for TC: 200 mg/dL; upper limit of normal for TGs: 150 mg/dL; upper limit of normal for LDL-C: 150 mg/dL; lower limit of normal for HDL-C: 45 mg/dL; and upper limit of normal for CEA: 5 ng/mL). The primary endpoint of the study was overall survival (OS). ROC analysis was performed to assess the prognostic significance of the proportional parameters presented. The cut-off values for TC/HDL-C and TGs/HDL-C were determined based on the results of the ROC analysis.

The right colon was considered as the segment from the cecum to the splenic flexure, and the left colon was considered as the segment from the splenic flexure to the rectum. Cancer stage was classified as I, II, III, and IV according to the 7th edition TNM staging system of the American Joint Committee on Cancer (AJCC). Overall survival (OS) was defined as the time from diagnosis to death.

The study was approved by the Kartal Dr. Lütfi Kırdar City Hospital Ethics Committee (number: 2024/010.99/5/17, approval date: 28 June 2024). This study was conducted in accordance with the Declaration of Helsinki.

### 2.2. Statistical Analysis

Data were statistically evaluated using the SPSS 22.0 software (SPSS Inc., Chicago, IL, USA). Chi-square and Fisher exact tests were used for comparative data. Numerical variables between two independent groups were analyzed with the Student *t*-test for normally distributed data or with the Mann–Whitney U test when data did not conform to a normal distribution. The effect of clinical and pathological features on OS was estimated using a Kaplan–Meier analysis. Survival predictors were analyzed using a multivariate Cox regression analysis. A two-sided *p*-value < 0.05 was considered statistically significant.

## 3. Results

### 3.1. Patient Characteristics

Of the 126 patients included, 46 (36.5%) were female, and 80 (63.5%) were male. Of the patients, 63 (50%) were younger than 65, while 63 (50%) were 65 years or older. Forty-five patients (35.7%) had right-sided tumors, and 81 patients (64.3%) had left-sided tumors. At the time of diagnosis, 71 patients (56.3%) were in early-stage (stage 1, 2, and 3), and 55 patients (43.7%) were in metastatic (stage 4) disease. The number of patients with CEA levels below the laboratory cut-off value of 5 ng/mL was 72 (57.1%), while the number of patients with CEA levels of 5 ng/mL and above was 54 (42.9%). The median follow-up time was 8 months (range: 0.56–130). The demographic and clinical data of the patients are shown in Table 1.

### 3.2. The Lipid Profiles of the Patients

When the patient distributions were examined according to the determined cut-off values, the laboratory values of the patients were below the determined values for LDL-C, TGs, and TC in the majority (>50%) (Table 2a). The trend was similar in tumors located in the right and left colons. Cut-off values were calculated as 4.16 for TC/HDL-C and 2.97 for TGs/HDL-C using an ROC analysis. When examined according to proportional parameters, there was a tendency for TGs/HDL-C and TC/HDL-C to be below the threshold values determined in patients with tumors located in the right colon and above the threshold values determined in patients with tumors located in the left colon. However, there was a tendency for HDL-C to be below the laboratory threshold value of 45 mg/dL in patients with tumors located in the left colon and to be 45 mg/dL and above in patients with tumors located in the left colon.

When lipid parameters were compared in terms of median values, HDL-C and TGs were found to be higher in patients with tumors located in the right colon than in patients with tumors located in the left colon, while LDL-C and TC were found to be higher in patients with tumors located in the left colon than in patients with tumors located in the right colon (Table 2b).

### 3.3. Lipid Parameters and Survival Analysis

**Figure 1:**
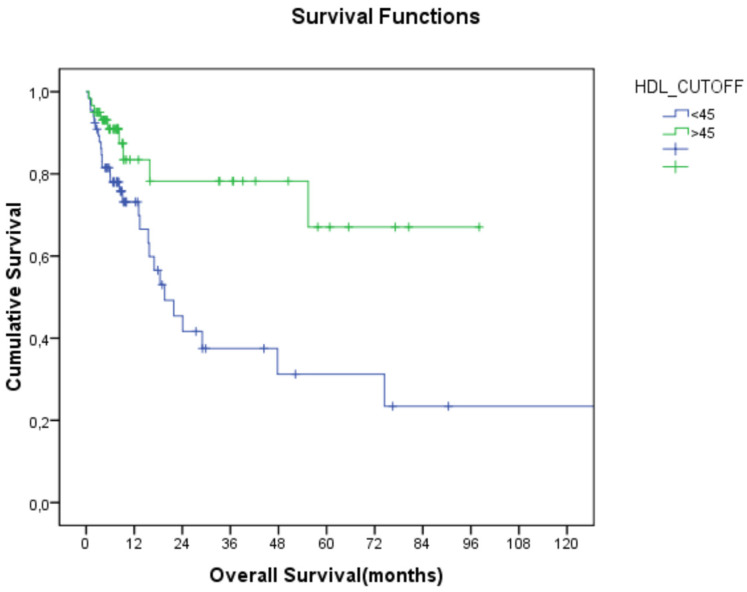
Relationship between HDL-C and overall survival (HDL-C: high-density lipoprotein cholesterol).

**Figure 2:**
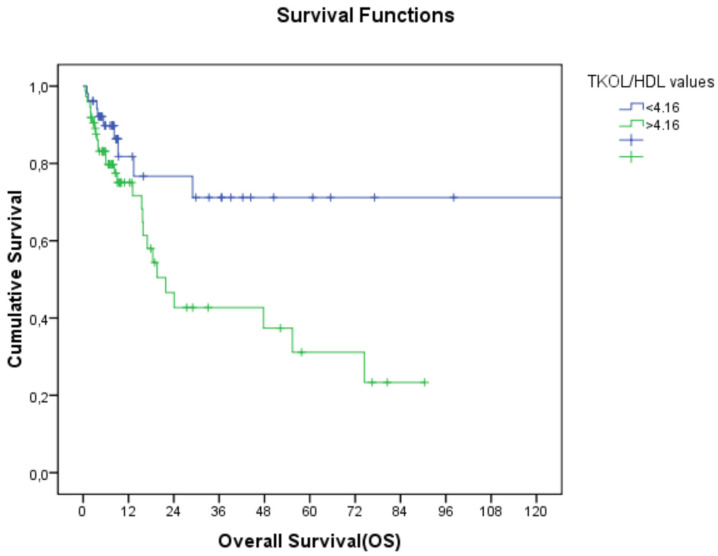
Relationship between TC/HDL-C and overall survival (TC/HDL-C: total cholesterol/high-density lipoprotein cholesterol).

The relationship between lipid parameters and overall survival (OS) was examined by stage and tumor location. HDL-C levels were found to be significantly associated with OS, regardless of stage, with higher HDL-C and lower TC/HDL-C levels being linked to better OS (19.9 months vs. NR, *p*: 0.004 for HDL-C, and 21 months vs. 130 months, *p*: 0.016 for TC/HDL-C; Table 3; Figure 1 and Figure 2).

When the patients were analyzed by early and late stages, HDL-C maintained its significance for early stages, and TC/HDL-C remained significant for advanced stages (*p*: 0.04 for HDL-C and *p*: 0.03 for TC/HDL-C; Table 4).

When the relationship between tumor localization and lipid parameters was evaluated statistically in terms of OS, HDL-C being 45 mg/dL and above and TC/HDL-C being 4.16 and above were statistically significant in tumors located in the left colon. For tumors located in the right colon, LDL-C being 150 mg/dL and below showed a statistically significant correlation in terms of OS (Table 5).

Factors predicting OS in the univariate analysis included age, stage, HDL-C, TC/HDL-C, and CEA (Table 6).

Parameters found to be significant in the univariate analysis were evaluated in a multivariate analysis. The multivariate analysis confirmed that age, stage, HDL-C, and TC/HDL-C remained significantly associated with OS (Table 7).

## 4. Discussion

Colorectal cancer (CRC) remains a major global health challenge, with its incidence continuing to rise in several regions, particularly in countries with previously low-risk populations. In our study, we aimed to investigate the relationship between blood lipid parameters, tumor location, and survival outcomes in CRC patients. Our findings suggest a complex interaction between lipid profiles, tumor location, and survival, providing new insights into the role of dyslipidemia in CRC prognosis.

### 4.1. Lipid Profiles and Overall Survival

One of the key findings of our study was the association between higher levels of HDL-C and better overall survival (OS) in CRC patients, regardless of tumor stage. This is consistent with previous studies that have suggested HDL-C may have protective effects against cancer progression, possibly due to its anti-inflammatory properties and its role in removing excess cholesterol from the bloodstream, which can otherwise contribute to tumor progression [26,27]. Moreover, higher HDL-C levels were associated with significantly improved OS in patients with early-stage disease, emphasizing the potential benefit of maintaining healthy lipid levels in the early stages of CRC. This finding supports the hypothesis that HDL-C may have a favorable impact on prognosis by limiting inflammation and promoting anti-tumor immunity, which are critical factors in early cancer progression.

Conversely, lower levels of HDL-C and higher TC/HDL-C ratios were associated with poorer survival outcomes, particularly in patients with advanced-stage disease. The TC/HDL-C ratio has been proposed as a potential prognostic marker in various cancers, including CRC, reflecting a more pro-inflammatory lipid environment that could contribute to tumor growth and metastasis [28,29].

### 4.2. Tumor Location and Survival

Our study also found that patients with left-sided tumors had higher TC/HDL-C ratios, which could suggest a more aggressive tumor biology in these patients, in line with previous studies that have observed different molecular characteristics between right- and left-sided CRC tumors [9,10]. The differential impact of lipid ratios on survival based on tumor laterality highlights the importance of tumor location in determining the prognostic value of lipid profiles in CRC.

Our study also found that patients with right-sided tumors had higher HDL-C levels and lower LDL-C levels, which could be indicative of a distinct metabolic environment in right-sided CRC. This is intriguing, as right-sided colon cancers have been historically associated with worse prognosis compared to left-sided cancers, possibly due to differences in tumor biology, immune microenvironment, and metabolic disturbances [11,12,13,14,15]. The higher HDL-C levels observed in right-sided tumors may reflect compensatory mechanisms in response to the local inflammation, although further studies are needed to elucidate the precise relationship between lipid metabolism and right-sided CRC biology.

In terms of lipid parameters, we also observed that patients with elevated LDL-C and TC levels had poorer survival outcomes, particularly in those with left-sided tumors. Elevated LDL-C is known to contribute to tumor progression through various mechanisms, including the activation of reactive oxygen species (ROS) and the MAPK signaling pathway, both of which play pivotal roles in colon cancer cell proliferation and metastasis [17]. This finding further supports the potential role of LDL-C as a mediator of cancer progression, reinforcing the importance of managing dyslipidemia in CRC patients.

### 4.3. Clinical Implications

The results of our study are consistent with previous research that has highlighted the role of lipid metabolism in cancer progression, but they also add new insights into the relationship between lipid profiles and tumor location in CRC. Our findings suggest that lipid metabolism may not only affect CRC prognosis in general but could also be influenced by the site of the tumor. Lipid parameters, such as HDL-C and TC/HDL-C ratios, could potentially be used as part of a prognostic panel to guide clinical decision making in CRC patients. However, due to the observational nature of our study, further prospective research with larger sample sizes is needed to validate these findings and explore the underlying mechanisms driving the observed associations.

### 4.4. Limitations and Future Directions

One limitation of our study is its retrospective design, which relies on data from a single institution, which may introduce selection bias. The exclusion of patients on lipid-lowering medications may have limited the generalizability of our findings, as these drugs can alter lipid levels and potentially affect cancer progression. Additionally, while we focused on lipid profiles and survival, the molecular and genetic factors underlying CRC progression, including microsatellite instability and mutations in key oncogenes (e.g., KRAS, BRAF), were not considered. These factors could interact with lipid metabolism to influence tumor behavior and prognosis. Another limitation of our study is the inclusion of patients with rectal cancer, despite the known differences in the biology and treatment of rectal cancer compared to other colorectal cancers. However, in this study, which aimed to evaluate the relationship between changes in lipid parameters and tumor location, it was believed that excluding patients with rectal cancer could lead to misleading conclusions. Another limitation of our study is the short follow-up period. We know that this situation may affect OS data.

Future studies should incorporate a more comprehensive approach, including genetic and molecular analyses, to better understand the interplay between lipid metabolism and CRC. Longitudinal studies with larger cohorts are needed to confirm the role of lipid parameters as reliable biomarkers for CRC prognosis, particularly in metastatic disease. Investigating the impact of lipid-lowering therapies, such as statins, in CRC treatment may also yield valuable insights.

## 5. Conclusions

In conclusion, our study provides evidence that lipid parameters, particularly HDL-C and TC/HDL-C ratios, are significantly associated with survival outcomes in CRC patients. The relationship between lipid profiles and tumor location underscores the importance of considering both metabolic factors and tumor biology when assessing prognosis in CRC. Further research is needed to refine the understanding of how lipid metabolism influences CRC progression and to explore whether lipid-modifying interventions could offer therapeutic benefits in CRC management.

While our study points to the influence of lipid metabolism on CRC prognosis, further investigation into the underlying mechanisms through advanced molecular and genetic analyses is necessary. Additionally, randomized controlled trials evaluating the therapeutic benefits of lipid-lowering interventions, such as statin therapy, in CRC treatment are warranted. Future prospective studies may provide further clarification on the role of lipid parameters in CRC prognosis.

## Figures and Tables

**Table 1:** Demographic and clinical characteristics of the patients (descriptive statistics analyses).

	*n* (%)
**Age**	
<65	63 (50%)
≥65	63 (50%)
**Gender**	
Female	46 (36.5%)
Male	80 (63.5%)
**ECOG**	
0	81 (64.3%)
1	31 (24.6%)
2	14 (11.1%)
**Localization**	
Right colon	45 (35.7%)
Left colon	81 (64.3%)
**Stage**	
Early stage (I, II, and III)	71 (56.3%)
Advanced stage (IV)	55 (43.7%)
**CEA**	
˂5 ng/mL	72 (57.1%)
≥5 ng/mL	54 (42.9%)

**Table 2:** (**a**) Lipid profiles of the patients according to cut-off values (descriptive statistics analyses with crosstabs). TC: total cholesterol, TGs: triglycerides, LDL-C: low-density lipoprotein cholesterol, HDL-C: high-density lipoprotein cholesterol, TC/HDL-C: total cholesterol/high-density lipoprotein cholesterol, TGs/HDL-C: triglycerides/high-density lipoprotein cholesterol, n: number. (**b**) Lipid parameters according to tumor localization (descriptive statistics analyses with crosstabs). TC: total cholesterol, TGs: triglycerides, LDL-C: low-density lipoprotein cholesterol, HDL-C: high-density lipoprotein cholesterol, *n*: number.

(a)				
Lipid Parameter	Cut-Off	Total, *n* (%)	Left Colon, *n* (%)	Right Colon, *n* (%)
**TC** (mg/dL)	>200	47 (37.9)	33 (40.7)	14 (31.1)
	≤200	79 (62.7)	48 (59.3)	31 (68.9)
**TGs** (mg/dL)	>150	46 (36.5)	29 (35.8)	17 (37.8)
	≤150	80 (63.5)	52 (64.2)	28 (62.2)
**LDL-C** (mg/dL)	>150	27 (21.4)	22 (27.2)	5 (11.1)
	≤150	99 (78.6)	59 (72.8)	40 (88.9)
**HDL-C** (mg/dL)	˂45	66 (52.4)	45 (55.6)	21 (46.7)
	≥45	60 (47.6)	36 (44.4)	24 (53.3)
**TC/HDL-C**	˂4.16	52 (41.3)	27 (33.3)	25 (55.6)
	≥4.16	74 (58.7)	54 (66.7)	20 (44.4)
**TGs/HDL-C**	˂2.97	61 (48.4)	37 (45.7)	24 (53.3)
	≥2.97	65 (51.6)	44 (54.3)	21 (46.7)
**(b)**				
**Lipid Parameters**		**Left Colon (*n* = 81)** (median (min–max))		**Right Colon (*n* = 45)** (median (min–max))
**HDL-C** (mg/dL)		42 (17–71)		45 (13–80)
**LDL-C** (mg/dL)		122 (60–257)		93.5 (33–271)
**TGs** (mg/dL)		130.5 (61–639)		136 (29–431)
**TC** (mg/dL)		196 (119–318)		172 (77–304)

**Table 3:** Relationship between lipid parameters and overall survival regardless of stage (Kaplan– Meier analyses). TC: total cholesterol, TGs: triglycerides, LDL-C: low-density lipoprotein cholesterol, HDL-C: high-density lipoprotein cholesterol, TC/HDL-C: total cholesterol/high-density lipoprotein cholesterol, TGs/HDL-C: triglycerides/high-density lipoprotein cholesterol, OS: overall survival.

Lipid Parameter	Cut-Off	OS	*p*-Value
**TC** (mg/dL)	>200	74	
	≤200	21	0.5
**TGs** (mg/dL)	>150	47	
	≤150	53	0.9
**LDL-C** (mg/dL)	>150	24	
	≤150	74	0.6
**HDL-C** (mg/dL)	˂45	19.9	
	≥45	NR	**0.004**
**TC/HDL-C**	˂4.16	130	
	≥4.16	21	**0.016**
**TGs/HDL-C**	˂2.97	28	
	≥2.97	55	0.98

**Table 4:** Relationship between lipid parameters and overall survival in early- and advanced-stage disease (Kaplan–Meier analyses). TC: total cholesterol, TGs: triglycerides, LDL-C: low-density lipoprotein cholesterol, HDL-C: high-density lipoprotein cholesterol, TC/HDL-C: total cholesterol/high-density lipoprotein cholesterol, TGs/HDL-C: triglycerides/high-density lipoprotein cholesterol, OS: overall survival, NR: not reached, *n*: number.

		Early Stages (Stage 1, 2, and 3) (*n* = 71)		Advanced Stage (Stage 4) (*n* = 55)	
Lipid Parameter	Cut-Off	OS	*p*-Value	OS	*p*-Value
**TC** (mg/dL)	>200	NR		16.9	
	≤200	130	0.8	8.2	0.58
**TGs** (mg/dL)	>150	130		19.5	
	≤150	NR	0.98	15.5	0.83
**LDL-C** (mg/dL)	>150	55		15.7	
	≤150	13	0.50	9.2	0.80
**HDL-C** (mg/dL)	˂45	74		15.5	
	≥45	NR	**0.04**	NR	0.09
**TC/HDL-C**	˂4.16	130		NR	
	≥4.16	74	0.41	15	**0.03**
**TGs/HDL-C**	˂2.97	NR	0.96	13.1	0.86
	≥2.97	130		18.4	

**Table 5:** Relationship between lipid parameters and overall survival according to left colon and right colon localization (Kaplan–Meier analyses). TC: total cholesterol, TGs: triglycerides, LDL-C: low-density lipoprotein cholesterol, HDL-C: high-density lipoprotein cholesterol, TC/HDL-C: total cholesterol/high-density lipoprotein cholesterol, TGs/HDL-C: triglycerides/high-density lipoprotein cholesterol, OS: overall survival).

		Left Colon (*n* = 81)		Right Colon (*n* = 45)	
Lipid Parameter	Cut-Off	OS (Months)	*p*-Value	OS (Months)	*p*-Value
**TC** (mg/dL)	>200	24		21.8	
	≤200	47.7	0.26	130	0.83
**TGs** (mg/dL)	>150	24		130	
	≤150	47.7	0.79	28	0.50
**LDL-C** (mg/dL)	>150	55.4		13.1	
	≤150	47.7	0.36	130	**0.018**
**HDL-C** (mg/dL)	˂45	19.5		21	
	≥45	NR	**0.01**	NR	0.11
**TC/HDL-C**	˂4.16	NR		130	
	≥4.16	19.5	**0.05**	21	0.26
**TGs/HDL-C**	˂2.97	NR		28	
	≥2.97	47.7	0.94	130	0.067

**Table 6:** Univariate analysis of parameters affecting overall survival using Cox regression analyses (TC: total cholesterol, TGs: triglycerides, LDL-C: low-density lipoprotein cholesterol, HDL-C: high-density lipoprotein cholesterol, TC/HDL-C: total cholesterol/high-density lipoprotein cholesterol, TGs/HDL-C: triglycerides/high-density lipoprotein cholesterol, CEA: carcinoembryonic antigen, HR: hazard ratio, CI: confidence interval).

	HR	*p*-Value	CI
**Age** (<65 vs. ≥65)	2.82	**0.004**	1.40–5.90
**Gender** (female vs. male)	1.37	0.39	0.66–2.83
**Localization** (left vs. right)	0.84	0.61	0.42–1.65
**Stage** (early vs. advanced)	0.11	**<0.01**	0.42–1.28
**HDL-C** (mg/dL)	0.34	**0.006**	0.16–0.74
**LDL-C** (mg/dL)	0.84	0.66	0.39–1.79
**TGs** (mg/dL)	1.02	0.94	0.51–2.04
**TC** (mg/dL)	1.20	0.59	0.60–2.41
**TGs/HDL-C**	1.00	0.98	0.52–1.93
**TC/HDL-C**	2.45	**0.02**	1.15–5.20
**CEA** (<5 vs. ≥5)	2.89	**0.003**	1.42–5.89

**Table 7:** Multivariate analysis of parameters affecting overall survival using Cox regression analyses (HDL-C: high-density lipoprotein cholesterol, TC/HDL-C: total cholesterol/high-density lipoprotein cholesterol, CEA: carcinoembryonic antigen, HR: hazard ratio, CI: confidence interval).

	HR	*p*-Value	CI
**Age** (<65 vs. ≥65)	2.24	**0.002**	1.11–4.53
**Stage** (early vs. advanced)	7.96	**<0.01**	3.05–20.81
**HDL-C** (mg/dL)	0.38	**0.01**	0.17–0.81
**TC/HDL-C**	1.22	0.05	0.47–3.16
**CEA** (<5 vs. ≥5)	1.77	0.12	0.85–3.69

## Data Availability

Data is available upon request to correspondence author.
